# A Modified Method for Whole Exome Resequencing from Minimal Amounts of Starting DNA

**DOI:** 10.1371/journal.pone.0032617

**Published:** 2012-03-05

**Authors:** Iwanka Kozarewa, Juan Manuel Rosa-Rosa, Christopher P. Wardell, Brian A. Walker, Kerry Fenwick, Ioannis Assiotis, Costas Mitsopoulos, Marketa Zvelebil, Gareth J. Morgan, Alan Ashworth, Christopher J.

**Affiliations:** 1 The Breakthrough Breast Cancer Research Centre, The Institute of Cancer Research, London, United Kingdom; 2 Haemato-Oncology Research Unit, The Institute of Cancer Research, Sutton, United Kingdom; 3 Cancer Research UK, Gene Function Group, The Institute of Cancer Research, London, United Kingdom; Auburn University, United States of America

## Abstract

Next generation DNA sequencing (NGS) technologies have revolutionized the pace at which whole genome and exome sequences can be generated. However, despite these advances, many of the methods for targeted resequencing, such as the generation of high-depth exome sequences, are somewhat limited by the relatively large amounts of starting DNA that are normally required. In the case of tumour analysis this is particularly pertinent as many tumour biopsies often return submicrogram quantities of DNA, especially when tumours are microdissected prior to analysis. Here, we present a method for exome capture and resequencing using as little as 50 ng of starting DNA. The sequencing libraries generated by this minimal starting amount (MSA-Cap) method generate datasets that are comparable to standard amount (SA) whole exome libraries that use three micrograms of starting DNA. This method, which can be performed in most laboratories using commonly available reagents, has the potential to enhance large scale profiling efforts such as the resequencing of tumour exomes.

## Introduction

The advent of massively parallel sequencing technologies has enabled the rapid and cost effective resequencing of cancer genomes and exomes [1], [2], [3]. However, despite the constant improvement of NGS technologies, one limitation is the amount of starting material that is often required. For example, standard protocols for targeted resequencing of the human exome using sequence capture [4] require, on average, one to three µg of starting DNA. These sequence capture methods involve DNA fragmentation, end repair and A-tailing of the fragmented DNA followed by hybridization to RNA (in case of the Agilent SureSelect system) or DNA baits (in case of the NimbleGen or Illumina systems) that are designed to physically capture specific DNA sequences. The captured DNA is then eluted from the baits, purified using a biotin-based precipitation and then amplified by PCR to yield enough material for sequencing. In the analysis of many tissue biopsies, such as microdissected tumour material, microgram amounts of DNA are often problematic to obtain. Around 125 ng of genomic DNA is usually recovered from an 8 micron (µ) thick microdissected tumour biopsy and therefore 30–40 tumour sections are required to obtain sufficient DNA for deep sequencing. With the increasing use of small core biopsies [5] and the requirement to perform multiple types of analysis on a single sample (e.g. genetic, genomic, transcriptomic, proteomic and histological analysis), the limitations of DNA recovery from biopsies are exacerbated.

Efforts have already been made to modify existing NGS sample preparation methods so that they may be applied to submicrogram amounts of starting DNA. These have either focussed upon using whole genome amplified (WGA) material [6], or exploiting transposon-mediated sample preparation [7]. However, both of these methods have significant drawbacks. WGA can result in libraries with low complexity, the absence of some targeted regions in the final sequenced library and can also be slightly biased towards the capture of AT-rich sequences [6]. Similarly, the use of transposon-coupled target systems with submicrogram amounts of DNA results in sequencing libraries with much lower complexity and capture specificity than microgram-based libraries [7].

Because of these problems, we have developed a modification of the commonly used Agilent SureSelect capture method that is able to use as little as 50 ng of starting DNA. By benchmarking the performance of sequencing libraries generated using this procedure, Minimal Starting Amount Capture Method (MSA-Cap), against widely accepted criteria (uniformity of coverage, library complexity, capture specificity, etc.) we find that our exome capture method generates high quality data that is comparable with sequencing libraries generated using microgram amounts of starting material. Moreover, MSA-Cap uses standard reagents and can be easily implemented in any laboratory, giving it the potential for widespread application.

## Materials and Methods

### DNA

The following genomic DNA samples were used for this study:

- Normal DNA derived from an immortalized cell line of a Caucasian female. This sample is part of the HapMap collection [8], number NA12813.
- Peripheral blood DNA from a patient with plasma cell leukaemia (abbreviated throughout the text as PCL-tumour, ∼90% tumour cells). This sample harbours translocations (t(11;14) and t(12;17)) and copy number changes (del(6q), del(13q), del(12p), del(17p) and gain(6p)), identified using GeneChip Mapping SNP 6.0 array.
- Non-tumour, buccal swab DNA from the patient described above (abbreviated PCL-buccal swab).

### Ethics statement

Ethics approval for the use of these samples was obtained from the Royal Marsden Hospital under CCR3019, REC 08/H0806/98. Informed written consent was obtained from the patient.

### Standard Exome library preparation

For the generation of standard exome capture libraries, we used the Agilent SureSelect Target Enrichment protocol for Illumina paired-end sequencing library (v. 2.0.1, May 2010) together with 3 µg input DNA. In all cases, the SureSelect Human All Exon Version 1 (38Mb) probe set was used.

### Minimal Starting Amount Capture (MSA-Cap) library preparation

**Figure 1:**
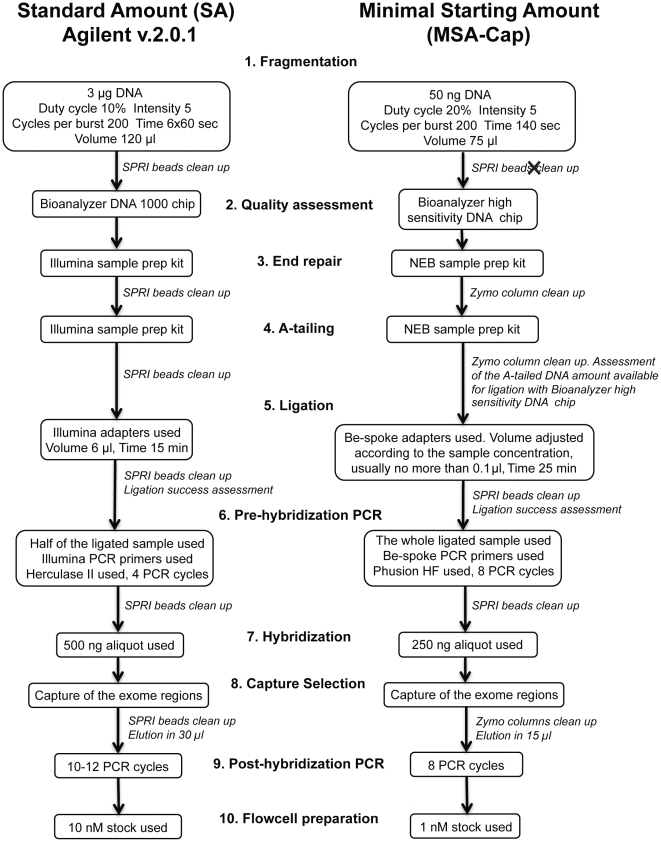
Flow chart comparing the standard Agilent Whole Exome Target Enrichment protocol (SA protocol, v. 2.0.1), and the Minimal Starting Amount Capture (MSA-Cap) protocol.

A step by step protocol for MSA-Cap is provided as Methods S1. In brief, MSA-Cap was performed as follows (numbers for each step refer to steps in Figure 1):

### (1) Fragmentation

Fragmentation of 50 ng of genomic DNA (estimated using a Qubit dsDNA High Sensitivity Assay) was performed using adaptive focused acoustic technology (AFA; Covaris) in a reaction volume of 75 µl (45 µl nuclease-free water plus 30 µl sample in Qiagen elution buffer EB). The fragmentation conditions used were: duty cycle 20%, intensity 5, cycle burst 200 and time 140 sec.

### (2) Assessment of fragmentation quality

The efficiency of fragmentation was assessed using Bioanalyzer High Sensitivity DNA assay.

### (3) End-repair

After fragmentation, samples were end-repaired using the New England Biolabs (NEB) sample preparation kit and protocol (NEB Next DNA Sample prep, E6000S), with incubation time of 30 minutes. Following end-repair, samples were purified using Zymo columns (DNA Clean&Concentrator kit-5, Zymo Research, USA) and eluted in 33 µl EB, pre-heated to 50°C.

### (4) A-tailing

After end-repair, a single dA was added to the 3′ end of each blunted, phosphorylated DNA template using Klenow Fragment DNA Polymerase (3′->5′ exo-) from the New England Biolabs (NEB) sample preparation kit (using an incubation time of 30 minutes). At the end of the incubation, the samples were purified with the DNA Clean&Concentrator kit-5 (Zymo Research, USA) and eluted in 12 µl EB.

### (5) Ligation

After A-tailing, DNA ligation was performed using the following adapters (* indicating phosphorothioate bond):

Top adapter 5′ ACACTCTTTCCCTACACGACGCTCTTCCGATC*T 3′ and
Bottom adapter 5′ GATCGGAAGAGCGGTTCAGCAGGAATGCCGAG 3′

Both oligonucleotides were HPLC purified and purchased from Sigma. The volume of the adapters was calculated as described in [9]. The ligation was conducted using the New England Biolabs (NEB) sample preparation kit and with an incubation time of 25 minutes. The samples were cleaned using solid-phase reversible immobilization (SPRI) magnetic beads and eluted in 96 µl nuclease-free water. Ligation efficiency was evaluated using the Bioanalyzer High Sensitivity DNA assay.

### (6) Pre-hybridization PCR

Once ligation had been assessed, the adapter ligated library was PCR amplified according to the following protocol: 92 µl of the adapter-ligated sample was mixed with 100 µl of stock 2× Phusion HF master mix and 4 µl of stock 25 µM PCR primer PE2.1 and 4 µl of stock 25 µM PCR primer PE2.2. The final 200 µl of sample was divided into four aliquots of 50 µl. Each was subjected to the following cycling conditions: step 1, 98°C for 2 min; step 2, 98°C for 20 sec; step 3, 65°C for 30 sec; step 4, 72°C for 30 sec; step 5, 72°C for 5 min; with steps 2 to 4 repeated 8 times in total. The amplified samples were purified using SPRI beads and eluted in 60 µl nuclease-free water. The amount of amplified material was estimated using a Bioanalyzer High Sensitivity assay.

The sequences of the PCR primers used above were as follows (* indicating phosphorothioate bond):

PE 2.1

5′ AATGATACGGCGACCACCGAGATCTACAC TCTTTCCCTACACGACGCTCTTCCGATC*T 3′

and PE 2.2

5′ CAAGCAGAAGACGGCATACGAGAT CGGTCTCGGCATTCCTGCTGAACCGCTCTTCCGATC*T 3′

Both oligonucleotides were HPLC-purified and purchased from Sigma.

### (7) Hybridization

From the purified sample, 250 ng of DNA was aliquoted into a 1.5-ml Eppendorf tube and then vacuum dried and resuspended in 3.4 µl nuclease-free water. Resuspended DNA was then mixed with hybridization buffers, blocking mixes, RNase block and 5 µl of SureSelect all exon capture library, according to the standard Agilent SureSelect Target Enrichment protocol. Hybridization to the exome capture baits was conducted at 65°C using heated thermal cycler lid option at 105°C for 24 hours on a Bio-Rad DNA Engine PCR machine.

### (8) Isolation of exomic DNA

Targeted DNA from the exomic regions was recovered using SureSelect magnetic beads (according to manufacturer's instructions) , washed thoroughly, eluted, purified using the using DNA Clean&Concentrator kit-5 and then collected in 15 µl nuclease-free water.

### (9) Post-hybridization PCR

The captured DNA was then amplified using the following protocol: 15 µl of the sample was mixed with 22.5 µl of nuclease-free water, 10 µl of 5× Herculase II reaction buffer, 0.5 µl dNTP mix, 1 µl Herculase II Phusion DNA polymerase and 1 µl SureSelect GA PCR primers. This reaction mix was subjected to the following cycling conditions: step 1, 98°C for 2 min; step 2, 98°C for 20 sec; step 3, 60°C for 30 sec; step 4, 72°C for 30 sec; step 5, 72°C for 5 min; step 6, 4°C on hold; with steps 2 to 4 repeated 8 times in total. The amplified samples were purified using SPRI beads and eluted in 60 µl nuclease-free water.

### (10) Flowcell preparation

Each sample was diluted or concentrated to 1.0 nM molarity and denatured following the standard Illumina protocol.

### Sequencing

All samples were sequenced on an Illumina Genome Analyzer IIX System, using 2×76 bp reads. Each sequencing run yielded ∼90% purity filtered clusters, providing 8–10 GB of raw yield per sample (Table S1).

### Data generation pipeline

Paired end short reads were generated with the Illumina OLB 1.8 and CASAVA 1.7 software pipelines using default parameters except for the removal of the first and last cycle from each read. The first cycle read is removed as a photobleaching effect on this cycle can often lead to an incorrect base call. The last cycle read does not have phase correction, therefore is also prone to errors in base call. Reads were further filtered to remove low complexity regions (over 95% same base call) and reads with over 5% N base calls. PCR duplicates were subsequently removed, retaining only the top Phred scoring paired end short read in each case.

All data were deposited in the Sequence Read Archive at the European Molecular Biology Laboratory's European Bioinformatics Institute (EMBL-EBI). The study accession number is ERP001066 (http://www.ebi.ac.uk/ena/data/view/ERP001066).

### Alignment, detection of SNPs and indels and structural variation study

The alignment of short reads to the human genome (hg19) was performed using BWA [10] and variants assigned using the Unified Genotyper within the Broad Institute Genome Analysis Toolkit (GATK) version 1.0.5273 [11], [12]. We used the Ensembl human variation database version 56 and the Ensembl Perl API system (http://www.ensembl.org/info/docs/api/index.html) to assign candidate functions to variants.

### SNP genotyping

We used the GeneChip Mapping SNP 6.0 array (Affymetrix, High Wycombe, UK) to assign SNP genotypes, according to manufacturer's instructions. SNP genotypes and copy number profiles were generated using GTYPE and dChip, as previously described [13].

### Copy number profiling by deep sequencing

Copy number profiling was performed as described in [14].

## Results

### A modified exome capture method

To facilitate targeted resequencing from minimal amounts of starting DNA, we designed a method, Minimal Starting Amount Capture (MSA-Cap), that is able to utilize as little as 50 ng starting DNA (Figure 1, Methods S1). In general MSA-Cap uses the same workflow as the Agilent SureSelect capture procedure (DNA fragmentation, end-repair, A-tailing, TA-mediated adapter ligation, PCR amplification, hybridization, post hybridization PCR (Figure S1), but encapsulates critical modifications at a number of steps (Figure 1). These modifications achieve three goals: (1) to minimize loss of DNA material during washing steps, (2) to use as much material as possible during PCR amplification steps, and (3) to minimize the amount of library DNA required for hybridization and flowcell preparation.

To minimise loss of DNA during washing steps we used the following modifications:

- An optimised DNA shearing protocol that enabled 50 ng DNA to be fragmented in 75 µL volume (Figure 1 step 1). This optimised protocol abolished the requirement for DNA to be concentrated after fragmentation, a step that can often lead to loss of ∼20% of material.
- Where DNA purification steps were absolutely required, we used the DNA Clean&Concentrator kit-5 containing Zymo clean columns (Zymo Research, USA) rather than more commonly used Qiagen columns or solid-phase reversible immobilization (SPRI) magnetic beads (Figure 1 steps 3 to 4, 4 to 5 and 8 to 9). In general we found Zymo columns to recover 27% more material than Qiagen columns (Figure S2).

Our second goal was to use as much material as possible during amplification. To achieve this, the following modifications were made:

- At the adapter ligation step, we used a 20∶1 molar ratio of adapter: fragmented DNA instead of the more commonly used 10∶1 ratio (Figure 1, step 5). We reasoned that the use of this ratio would result in a higher yield of adapter ligated templates. In addition, the time course of the ligation reaction was extended to 25 minutes instead of 15 minutes to maximize the ultimate amount of ligated material.
- The use of additional PCR cycles at the pre-hybridization step (Figure 1, step 6) to obtain the DNA amount (250 ng) required for hybridization.
- Utilization of the entire DNA template at both the pre-hybridization and the post-hybridization PCR steps (Figure 1, steps 6 and 9).

Finally, to achieve the third goal which was to minimize the amount of DNA required for hybridization and flowcell preparation, we included the following modifications:

- The use of 250 ng of PCR product in the hybridization step rather than 500 ng. On average 250 ng DNA is equivalent to 9.26×10^11^×250 bp DNA fragments, which constitutes roughly 76,000 exomes.
- In the preparation of flowcells, we varied the volume of 10 mM Tris-Cl, pH 8.5 used during the denaturation step to enable larger volume of DNA to be used. This allowed us to reduce the amount of DNA required for efficient cluster generation from 10.0 nM to 1.0 nM.

In addition, to assess the success of each DNA modification, we used the Bioanalyzer DNA High Sensitivity assay (Agilent Technologies, Santa Clara, CA) rather than the more commonly utilized DNA 1000 assay (Agilent Technologies). The DNA 1000 assay uses, on average, 20 times more DNA than the High Sensitivity assay. We also included two additional quality control visualization steps. Firstly, we visualized samples immediately post-fragmentation to assess the efficiency of fragmentation (the percentage of the starting material sheared to the required size and the percentage loss of starting material). When fragmentation was unsuccessful, high molecular weight DNA fragments were carried through the sample preparation procedure. This reduced the sequencing quality by increasing the range of DNA fragment sizes in the final sequencing libraries.

Secondly, we used the High Sensitivity assay prior to ligation (Figure 1, step 5) to measure the amount of A-tailed sample and thus calculate the adapter volume to use to maintain 20∶1 (adapter: sample ratio during ligation). The latter ratio allowed us to simultaneously maximize our yield of fully ligated templates and minimize adapter dimers contamination.

### Benchmarking MSA-Cap sequencing libraries

To assess the quality of sequencing libraries generated with the MSA-Cap method we compared DNA sequence data generated from MSA-Cap libraries using 50 ng starting material, to those generated by the standard amount (SA) exon capture procedure using 3 µg starting DNA. We used three different sets of samples: (i) genomic DNA from a female HapMap sample (NA12813) for which single nucleotide variants (SNVs) had previously been identified [8], (ii) genomic DNA from a plasma cell leukaemia (PCL) tumour sample with known copy number variation (CNV) and SNV status, and (iii) normal (non-tumour) DNA from the same individual as in (ii), derived from a buccal swab. All samples were sequenced using 2×76 bp paired-end runs that in each case resulted in ∼9 Gb raw yield per library (Table S1). Initial inspection of the Intensity Versus Cycle (IVC) plots as well as the summary statistics produced by the Illumina pipeline revealed comparable metrics for all the libraries in terms of mappable yield, insert size, percent purity filtered reads and percent aligned reads (Table S1).

So as to compare the quality of SA libraries and MSA-Cap libraries, we compared sequencing data in terms of the frequency of reads mapped to the reference genome, the median depth of sequencing reads across the genome, and finally the coverage of sequencing data.

The frequency of reads mapped to the genome (% reads mapped) was similar for SA and MSA-Cap samples, irrespective of the sample type (Table 1). The median depth of coverage for the three sample sets (PCL-tumour, PCL-buccal swab and NA12813) in which MSA-Cap sample preparation was compared to SA was between 68 and 106 (Table 1). Regardless of difference in coverage between the different samples the percentage bases covered at least by one read (coverage at ≥1×) was similar for all samples and libraries (between 97% and 99%, Table 1). When coverage ≥20× was taken into account (usually required for reliable SNV calling), the percentage coverage was marginally lower for the PCL MSA-Cap libraries than for their respective SA libraries (Table 1, 3–4% lower) although this could be compensated by increasing sequencing depth.

**Table 1:** Target enrichment and sequencing quality metrics.

	PCL-tumour DNA		PCL-buccal Swab DNA		NA12813 DNA	
	SA	MSA-Cap	SA	MSA-Cap	SA	MSA-Cap
**% Reads mapped**	90%	92%	93%	95%	90%	93%
**Median depth of coverage**	98	98	106	94	68	76
**Coverage (≥1×)**	98%	97%	99%	99%	97%	99%
**Coverage (≥20×)**	88%	84%	92%	88%	79%	87%
**% Unique reads**	96%	89%	91%	83%	86%	78%
**% Reads on target**	56%	55%	60%	60%	57%	57%

**Figure 2:**
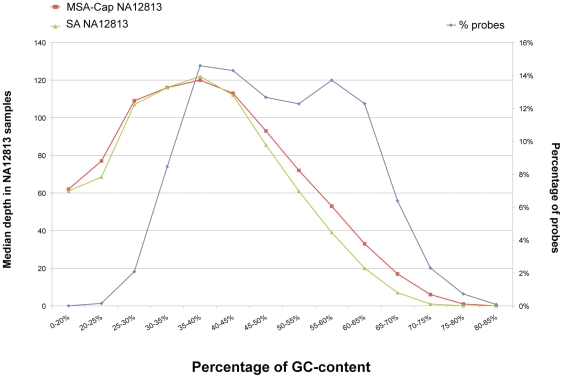
Capture efficiency of probes with different GC-content (percentage of GC bases from the total bases) for SA and MSA-Cap HapMap (NA12813) libraries. The efficiency is shown as median depth of coverage of probe regions with different GC percentage. The GC percentage range of the probes is shown on the x axis. The median depth of coverage for the probes in the HapMap sample, either SA or MSA-Cap libraries, is represented on the left y axis. The percentage of probes in each GC content is shown on the right y axis.

We found the capture efficiency using Agilent SureSelect Human All Exon kit version 1 to be dependent upon the GC-content of the targeted region, with regions of GC-content between 30% and 50% being preferentially captured and regions with GC-content above 70% having minimal representation in the final sequencing library (Figure 2). This GC-bias likely contributes to the non-uniformity of coverage and could explain the absence of some targeted GC-rich regions in the final datasets. However, we did not observe any difference in the GC-bias between SA- and MSA-Cap samples (Figure 2).

Another parameter that defines the success of whole exome capture and sequencing is library complexity, i.e. the percentage of reads that following mapping are found to have unique start sites. Low library complexity can result in exomic regions being absent from the final sequencing libraries and that unable to be used for data generation. The percentage of reads with unique start sites was marginally lower for all MSA-Cap libraries: 89% in MSA-Cap vs. 96% in SA for PCL-buccal swab, 83% in MSA-Cap vs. 91% in SA for PCL-tumour and 78% in MSA-Cap vs. 86% in SA for NA12813 (Table 1). Importantly, the decrease in number of reads with unique starts did not affect the capture specificity (percentage of reads that map on target) in MSA-Cap libraries. We found the latter parameter to be between 55–60% for all samples (Table 1).

Finally, the MSA-Cap library preparation method compared favourably to the standard (SA) method in respect to time required for sample preparation and the cost of sample preparation. In fact because of the replacement of SPRI bead purification with Zymo column steps and the removal of one additional purification step, the MSA-Cap sample preparation procedure was actually shorter by two hours than the standard procedure (Methods S1, Appendix 3). The cost of the MSA-Cap sample preparation was marginally higher (2%) than that of the SA library preparation.

### Genotype calling from MSA-Cap libraries

One of the major uses of DNA sequencing is the identification of genetic mutations. In order to assess the validity of MSA-Cap libraries we investigated the concordance between SNP calls made from deep sequenced MSA-Cap libraries with those made using an orthogonal technology, Affymetrix SNP6 DNA arrays.

Using an analysis of SNP calls in a PCL-tumour sample and a HapMap sample, we found MSA-Cap libraries to give highly concordant (>96%) SNP calls when compared to SNP6 genotypes (Table 2). The extent of agreement between MSA-Cap library SNP calls and corresponding SNP6 genotypes was also similar when SA libraries were used.

**Table 2:** Genotype concordance for SNP calls in a PCL-tumour sample and a HapMap (NA12813) sample.

	PCL-tumour		NA12813	
Method of preparation	SA	MSA-Cap	SA	MSA-Cap
**% Known SNPs miscalled by deep sequencing**	5%	4%	6%	5%
**% Known SNPs not called by deep sequencing**	0%	0%	4%	1%
**% Homozygous variants called as heterozygous by deep sequencing**	4%	3%	2%	2%
**Total SNP concordance of deep sequencing data with SNP6 array data**	99%	99%	93%	96%

For the PCL-tumour sample, the variants called by deep sequencing were compared to data from Affymetrix SNP 6.0 DNA array while for the HapMap sample published data were used for the comparison.

### Comparison of genome-wide variants identified by deep sequencing

**Figure 3:**
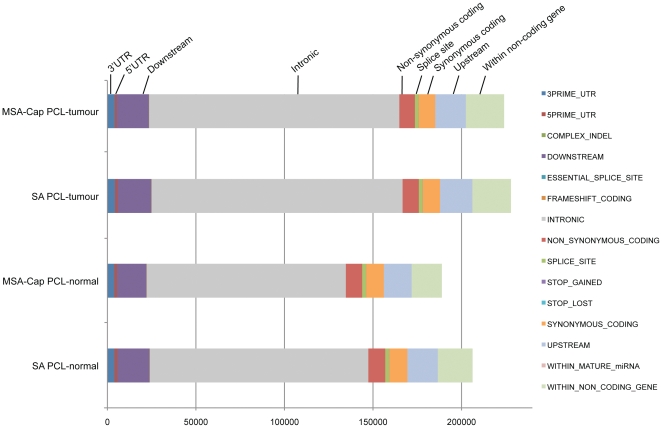
Mutational spectrum from SA and MSA-Cap libraries, prepared from PCL-tumour or PCL-buccal swab samples. Non-filtered variants were identified in each sample using Broad Institute Best Practice pipeline version 1.0.5273 upon comparison to human hg19 reference sequence and categorized according to putative gene effect. The cumulative number of variants for each library type is shown on the x axis and the sample and library type on the y axis. Variant categories, as assigned by the Ensembl database, are on the right side of the figure.

In addition to the comparison with SNP6 genotypes, we examined the quality of MSA-Cap libraries by assessing the similarity in mutational spectrum between MSA-Cap and SA libraries derived from the same genomic DNA sample. Here we used the GATK to call variants in a normal and tumour sample from a PCL patient. As shown in Figure 3, the mutational spectrum was very similar in all libraries, suggesting that MSA-Cap can deliver mutational data similar to the standard method of exome capture and resequencing.

**Figure 4:**
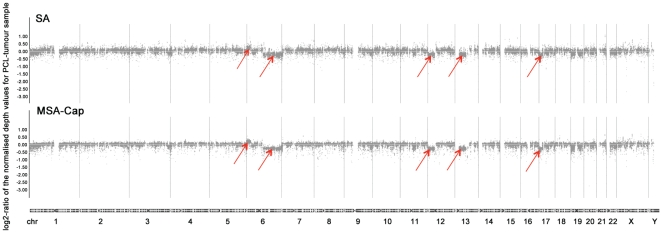
Copy number profiles estimated by deep sequencing of PCL-tumour samples, prepared using the SA- or MSA-Cap methods. Chromosomes 1 to 22 plus X and Y are shown on the x axis. The log_2_ ratio of the normalized by the normal depth values for the PCL-tumour libraries is represented on the y axis. Arrows point towards the detected by deep sequencing and previously identified by SNP6 array copy number changes: gain (6p), del (6q), del (12p), del (13q) and del (17p).

As part of this analysis, we also generated copy number profiles for a cancer genome from each library and compared these to each other as well as to the known copy number aberrations in the sample identified by Affymetrix DNA SNP6 array. The copy number profile from the MSA-Cap library compared favourably to the profile generated from the SA library (Figure 4) with the deletions in 6q, 12p, 13q, 17p and the gain in 6p clearly distinguishable.

## Discussion

The goal of this study was to develop a simple target enrichment protocol that could be used on DNA templates of less than 1 µg. Our method and the subsequent analysis of MSA-Cap libraries suggest that this is possible and that as little as 50 ng of DNA may be used as the starting amount. Given the limited amount of DNA often recovered from small samples (such as fine needle aspirates, for example), it is possible that this method will find considerable utility. Moreover, by using the Agilent Sure Select system as a basis for our adapted protocol, the reagents needed to perform MSA-Cap are widely available and the method useable by any laboratory. In our facility, the MSA-Cap library preparation method has also been used to prepare sequencing libraries from 44 clinical (tumour-matched normal multiple myeloma) samples (Walker et al., in preparation). The quality of the libraries obtained was consistent with the results presented in this study (Walker et al., in preparation).

Our protocol and the accompanying analysis provide a number of benefits when compared to other submicrogram DNA-based whole exome protocols. An alternative system, the transposon-based whole exome capture protocol (7), also uses 50 ng of starting DNA material. However, the enrichment and sequencing parameters described by Adey and colleagues [7] were as follows: 78% reads mapping, 47% of which on-target and 87% coverage ≥1×. These results did not compare favourably to our method, in which the percentage of reads mapping was over 14% higher, whilst the average coverage at 1× depth was 98% (Table 1). Importantly, the study of Adey and colleagues (7) did not examine variant concordance levels between deep sequencing and an orthogonal technology such as SNP arrays. Thus, it remains uncertain whether the transposon-based whole exome capture method can effectively deliver high-quality variant analysis.

In conclusion, MSA-Cap provides a straightforward method for the capture of exomic DNA (and potentially other targeted regions) in situations when template DNA is in short supply. As we have shown with the SNPs/indels and CNVs identification in matched tumour and normal sample pairs, one particular utility of this method will be in the analysis of tumour samples. We also note that the ability of our method to deliver reliable variant calls from 50 ng of starting DNA will also allow the exomic resequencing of multiple different microdissected fractions from the same tumour, and thus an increasing understanding of intra-tumoural genetic heterogeneity [15].

## Supporting Information

Methods S1 Minimal Starting Amount Sample Preparation Protocol (MSA-Cap).
(PDF)

Figure S1 Overview flowchart of the steps involved in target enrichment and sequencing.
(PDF)

Figure S2 Bioanalyzer High Sensitivity DNA Assay showing extent of recovery of fragmented 50 ng commercial (Novagen) DNA material using Zymo columns (two independent samples in A, B) and Qiagen column (C). The DNA was sheared using the MSA-Cap fragmentation conditions and purified according to manufacturers' instructions. In each case 1 l of the product was loaded. 2.04 ng/µl or 1.69 ng/µl were recovered using Zymo columns (A, B) in contrast to 1.36 ng/µl when using Qiagen column (C).
(PDF)

Table S1 Sequencing statistics for a PCL-tumour sample, PCL-buccal swab sample and a HapMap (NA12813) sample.
(PDF)
