# Correction: A Modified Method for Whole Exome Resequencing from Minimal Amounts of Starting DNA

**DOI:** 10.1371/annotation/5b071c93-9fa8-4be6-9cbd-95aead146305

**Published:** 2012-04-03

**Authors:** Iwanka Kozarewa, Juan Manuel Rosa-Rosa, Christopher P. Wardell, Brian A. Walker, Kerry Fenwick, Ioannis Assiotis, Costas Mitsopoulos, Marketa Zvelebil, Gareth J. Morgan, Alan Ashworth, Christopher J. Lord

The corresponding author's last name was cut off in the online version of the article. The corresponding author's full name is Christopher J. Lord.

