# Supplementary material for: A Modified Method for Whole Exome Resequencing from Minimal Amounts of Starting DNA
Source: PLoS One. 2012 Mar 5;7(3):e32617. doi: 10.1371/journal.pone.0032617 (PMC3293839; doi:10.1371/journal.pone.0032617)
Supplement: Methods S1 — Minimal Starting Amount Sample Preparation Protocol (MSA-Cap). (PDF) [file pone.0032617.s001.pdf]

Methods S1

Minimal Starting Amount Sample Preparation Protocol (MSA-Cap)

1. Methods.

1.1. Fragmentation.

- Start from 50-60 ng DNA in 10-30 µl Elution buffer (EB) or TE buffer (10mM Tris, pH 7.5/ 1 mM EDTA.
- Add EB buffer to make the final volume of 75µl.
- Transfer the samples to 130µl Crimp-Cap microTUBE with AFA fiber.
- Shear the samples for 140 sec with the following program:

  Duty cycle: 20%

  Intensity: 5

  Cycle burst: 200

  Power: 37W

  Temperature: 7°C

  Mode: freq sweeping

- Transfer the samples into 1.5 ml RNase-free non-stick tubes.
- Check the quality of fragmentation by running the samples on an Agilent High Sensitivity DNA chip. A 150-250 bp smear should be detected with 70-90% of the initial DNA amount recovered.

PAUSE POINT: fragmented samples can be stored at -20°C for up to 4 weeks.

1.2. End-repair.

- For each sample, prepare the following master mix from the NEBNext DNA Sample Prep Reagent Set 1 with 10% excess for pipetting errors:

10μl	Phosphorylation buffer
4μl	10 mM dNTP mix
5μl	3U/μl T4 DNA polymerase
1 μl	5U/μl Klenow DNA polymerase
5μl	10U/μl T4 PNK

- Mix and aliquot 25 μl of master mix into each sample tube containing 75 μl of sheared DNA. Mix well by pipetting up and down.
- Incubate for 30 minutes in a thermal cycler at 20°C. Switch off heated lid.
- Purify end-repaired DNA using a Zymo DNA Clean & Concentrator™-5 column following the manufacturer's instructions and using 5:1 ratio of DNA Binding Buffer: sample volume.
- Elute DNA in 33 μl EB pre-heated to 50°C.

PAUSE POINT: end-repaired samples can be stored at -20°C for couple of days.

1.3. A-tailing.

- For each sample, prepare the following master mix from the NEBNext DNA Sample Prep Reagent Set 1 kit with a 10% excess:

5μl	10x NEB buffer 2
10μl	1 mM dATP
3μl	5U/μl Klenow fragment (3' to 5' exo minus)

- Aliquot 18 μl of mix to each sample and mix well by pipetting.
- Incubate at 37°C for 30 minutes in a thermal cycler with the heated lid set at 37°C ±5°C.
- Purify the A-tailed DNA using a Zymo DNA Clean & Concentrator™-5 column following the manufacturer's instructions and using 5:1 ratio of DNA Binding Buffer : sample volume.
- Elute DNA in 12 μl EB pre-heated to 50°C.
- Check the quantity of the recovered A-tailed samples by running them on an Agilent High Sensitivity DNA chip. Use the 'Region' function to calculate the DNA quantity in ng/μl.

1.4. Ligation.

- Using the formula below, calculate the moles A-tailed samples. Use a molar ratio of 20:1 of adapters: A-tailed sample. The adapters are prepared as described in Appendix 1.

$$\text{Moles} = \frac{\text{DNA mass in g}}{\text{average length of the fragmented DNA X 650 (average weight of a base pair)}}$$

- For each sample, prepare the following master mix with a 10% excess:

25 μl	2x quick ligation buffer (from the NEB kit)
xμl	Adapter mix (1:10 dilution in T4 ligase buffer with 10mM ATP)*
10-xμl	Water

5μl	2000U/μl T4 DNA ligase
40μl	TOTAL

*The volume adapters required for 20:1 adapter: sample ratio.

The adapter mix is prepared as described in Appendix 1.

- Mix and add 40 μl of mix to sample (total volume 50 μl).
- Incubate at 20°C for 25 minutes.
- Purify the samples using AMPure SPRI beads (see Appendix 2). Elute the samples in 96ul nuclease-free water.

PAUSE POINT: purified or unpurified ligations can be stored at -20°C for up to 1 week.

1.5. Pre-hybridization PCR.

- For each sample, prepare the following master mix, plus a 10% excess:

4 μl	25 μM primer PE2.1
4 μl	25 μM primer PE2.2
100 μl	2x Phusion HF master mix
92 μl	ligated sample

- You will need to use the entire ligated product. Check whether the volume is 92 μl and adjust to 92 μl accordingly.
- Aliquot each sample into 4 0.2ml thin wall PCR tubes (50 μl in each) and perform the following cycling conditions.

98°C for 2 minutes

8 cycles of:	98°C for 20 seconds
	65°C for 30 seconds
	72°C for 30 seconds
Then	72°C for 5 minutes
	4°C indefinitely

- Purify the PCR product using AMPure SPRI beads as described in Appendix 2, except:
  - o Combine all 4 PCRs into one tube and add 360 μl beads.
  - o Elute DNA in 40 μl nuclease-free water.
- Check the quantity of the libraries obtained by running them on an Agilent High Sensitivity DNA chip. Use the 'Region' function to calculate the DNA quantity in ng/μl.

PAUSE POINT: purified or unpurified PCR reactions can be stored at -20°C for up to 6 weeks.

- Separate 250 ng of DNA library to a 1.5 ml RNase-free non-stick tube.
- Vacuum dry the samples at <45°C until dry.
- Resuspend the samples in 3.4 μl nuclease-free water overnight at 22°C.

1.6. Hybridisation

1.6.1. Preparation step.

- **A – Hybridisation Buffer**

Mix the components below (from the Agilent SureSelect kit) at room temperature to prepare the hybridization buffer:

**Reagent**	**x1**	**x12**
SureSelect Hyb #1	25μl	275μl
SureSelect Hyb #2	1μl	11μl
SureSelect Hyb #3	10μl	110μl
SureSelect Hyb #4	13μl	143μl
**Total**	**49μl**	**539μl**

If a precipitate forms warm the buffer at 65°C for 5 minutes.

- **B – Library Preparation**

In 0.2 ml PCR thin wall strip tubes prepare the following:

3.4 μl	250ng DNA library
2.5 μl	SureSelect block #1 (green cap)
2.5 μl	SureSelect block #2 (blue cap)
0.6 μl	SureSelect PE block #3 (brown cap)

Mix by pipetting.

- **C – Oligo Library Capture Mix**

In 0.2 ml thin wall PCR strip tubes prepare the following:

5 μl	SureSelect Oligo Capture library
1 μl	Nuclease-free water
1 μl	RNase block (purple cap, diluted 1:1 in water)

Place in fridge until ready to use.

1.6.2. Hybridization step.

Set a thermal cycler to the following temperatures with heated lid at 105°C:

95°C	5 minutes
65°C	indefinitely

- Place empty 0.2 ml strip tubes at either end of the PCR machine to prevent crushing tubes with samples in by the heated lid.
- Place the library preps (B) into column 3 and heat the samples to 95°C for 5 minutes followed by 65°C forever.
- Place the strip tubes labelled A in the PCR machine into column 2 and pipette 40 μl of Hybridisation buffer (A above) into each tube per sample.
- Incubate at 65°C for 5 minutes.
- Keeping the tubes in the PCR machine, place the library capture mix (C) in column 4 and heat at 65°C for 2 minutes.
- In the PCR machine transfer 13 μl of A (hyb buffer) to C (capture mix). Then transfer 9 μl of DNA library (B) to C (hyb+capture).
- Transfer entire contents of C (hyb+capture+library) to new 0.2 ml tubes, cap and incubate at 65°C for 24 hours with the heated lid at 105°C. Keep used strips either side of samples to prevent crushing of tubes.

1.7. Capture of the hybridized exome regions.

1.7.1 Magnetic Bead Preparation.

- Pre-warm SureSelect Wash Buffer #2 from the Agilent SureSelect kit at 65°C.

- Resuspend MyOne Streptavidin T1 Dynabeads and for each sample separate 50 μl to a 1.5 ml Eppendorf tube.
- Add 200 μl SureSelect binding buffer to each tube and mix by vortexing.
- Place the tube in a magnet and remove and discard the supernatant.
- Repeat wash with binding buffer for a total of 3 washes.
- Resuspend the beads in 200 μl SureSelect Binding buffer.

1.7.2 Hybrid Capture.

- Estimate and record the volume of each sample remaining after the 24 hour hybridisation.
- Add the hybridization mixture directly from the thermal cycler to the bead slurry and invert 3-5 times.
- Incubate the mixture on a rotator for 30 minutes at room temperature, ensuring the sample is mixing properly.
- Briefly centrifuge, place on a magnet stand and remove the supernatant.
- Add 500 μl SureSelect Wash Buffer #1 and vortex.
- Incubate beads at room temperature for 15 minutes.
- Place on a magnet, remove supernatant and add 500 μl pre-warmed at 65°C Wash Buffer #2 (1.6.1.) and vortex.
- Incubate at 65°C for 10 minutes.
- Place tube on magnet, remove supernatant and repeat wash with buffer #2 for a total of 3 washes.
- Ensure all buffer has been removed from beads.
- Mix the beads with 50 μl SureSelect Elution buffer by vortexing.

- Incubate at room temperature for 10 minutes.
- Place tube on magnet and transfer supernatant to a new 1.5 ml tube. The supernatant contains the captured DNA and the beads can be discarded.
- Add 50 μl SureSelect Neutralization buffer to the captured DNA.
- Purify the captured DNA using a Zymo column following the manifacturer's instructions and using 5:1 ratio of DNA Binding Buffer: sample volume.
- Elute DNA in 17 μl EB pre-heated to 50°C.

PAUSE POINT: purified captured DNA samples can be stored at -20°C for up to 1 week.

1.8. Post-hybridization PCR.

- Prepare one amplification reaction per sample, and include a negative control.

Nuclease-free water	22.5μl
5X Herculase II buffer	10.0μl
dNTP mix (25 mM each)	0.5μl
Herculase II Fusion DNA Pol	1.0μl
SureSelect GA PCR primers	1.0μl
**Total**	**35.0 μl**

- Add 35 μl of the master mix to each 15 μl library in 0.2 ml PCR tubes.
- Run the following PCR program:

98°C for 2 minutes

8 cycles of: 98°C for 20 seconds

60°C for 30 seconds

72°C for 30seconds

Then: 72°C for 5 minutes

4°C indefinitely

- Purify the PCR reaction using AMPure SPRI beads as in Appendix 2.
- Elute in 60 μl nuclease-free water.
- Assess the quantity of the final sequencing library using a High sensitivity DNA Bioanalyzer chip.

PAUSE POINT: the ready for sequencing libraries can be stored at -20°C for up to 6 months.

1.9. Flowcell preparation.

- Final sequencing libraries were adjusted to 1nM and denatured in 0.5N NaOH.

xµl*	Final sequencing library
1µl	2N NaOH
19ul-Xµl	EB
20µl	Total volume

*x is the volume of sample required to give 1nM molarity in 20µl.

- Libraries were incubated at room temperature for 5 minutes to denature.

- Dilution and remaining flowcell preparation were carried out according to manufacturer’s protocol for Paired-End Cluster Generation Kit v4 (Illumina).

2. Timing:

1. Sample fragmentation.	10 minutes per sample
2. End-repair, A-tailing and ligation.	6 hours
3. AMPure SPRI-beads purification.	50 minutes
4. Pre-hybridization and post-hybridization PCR.	
4.1. Set up	0.5 hour
4.2. PCR	0.5 hours
4.3. Clean up	40 minutes
5. Hybridization.	
5.1. Set up	1 hour
5.2. Duration	24 hours
6. Capture of the exome regions.	
6.1. Magnetic Bead Preparation	0.5 hour
6.2. Hybrid Capture	4 hours
7. Flowcell preparation	1 hour

3. Consumables.

- SureSelect Human All Exon Kit (Agilent Technologies UK, Edinburgh, UK, cat. no. G3362B)

- Human All Exon 50Mb Kit (Agilent Technologies UK, Edinburgh, UK, cat. no. G3370B)
- Agencourt AMPure SPRI beads (Beckman Coulter, Brea, CA cat. no A29152)
- QIAquick PCR purification kit (Qiagen, Valencia, CA cat. no. 28104)
- HPLC-purified indexing adapters (Sigma, St.Louis, MO)
- Crimp-Cap microTUBE with AFA fiber (Covaris, Woburn, MA cat. no. 520052)
- Agilent High Sensitivity DNA chip (Agilent Technologies UK, Edinburgh, UK, cat. no. 5067-4626)
- Agilent DNA 1000 chips (Agilent Technologies UK, Edinburgh, UK, CA cat. no. 5067-1504)
- RNase-free non-stick tubes 1.5ml (Applied Biosystems/Ambion, Austin, TX, cat. no. AM12450)
- DNA Clean & Concentrator™-5 (Zymo Research, Irvine, CA cat. No.D4013)
- 0.2ml Thin Wall Clear PCR Strip Tubes and Clear Strip Caps. (Axygen, Union City, CA PCR-0208-CP-C)
- NEBNext DNA Sample Prep Reagent Set 1 (NEB, Hitchin, Herts, UK cat. no. E6000S)
- Phusion® High-Fidelity PCR Master Mix with HF Buffer (Finnzymes Oy, Vantaa, Finland, cat. no.F-531S)
- Dynabeads MyOne Streptavidin T1 (Life Technologies Ltd, Paisley , UK cat. no. 656-01)
- Paired-End Cluster Generation Kit v4 (Illumina Inc, San Diego, CA, USA, cat. no. PE-203-4005)
- HPLC-purified Adapters (Sigma, St.Louis, MO)

Top_adapter 5' ACACTCTTTCCCTACACGACGCTCTTCCGATC*T 3'

*indicates phosphorothioate

Bottom_adapter 5' GATCGGAAGAGCGGTTCAGCAGGAATGCCGAG 3'

- HPLC-purified pre-hybridization PCR primers (Sigma, St.Louis, MO)

PE2.1 5'AATGATACGGCGACCACCGAGATCTACACTCTTTCCCTACACGACGCTCTTCCGATC*T

PE2.2

5'CAAGCAGAAGACGGCATACGAGAT CGGTCTCGGCATTCCTGCTGAACCGCTCTTCCGATC*T 3'

*indicates phosphorothioate

5. Appendix 1.

- Resuspend adapter oligos to a final concentration of 100 μM in water.
- Combine the following in one 0.2ml thin wall PCR tube:

100μM Top_adapter	20μl
100μM Bottom_adapter	20μl
T4 ligase buffer with 10mM ATP	5μl
T4 PNK	5μl

- Vortex, spin and place the tube in the thermal cycler.
- Use the following cycling conditions:

30 minutes at 37°C

Ramp PCR machine at 0.5ºC / sec to 97.5ºC

Hold at 97.5ºC for 150 seconds

Then 97.5ºC for 5 seconds and temp drop of (-) 0.1ºC per cycle for 775 cycles (i.e. decrease temperature from 97.5ºC by 0.1ºC every 5 seconds).

4°C indefinite

- Store at -20°C in 8μl aliquots.

6. Appendix 2.

AMPure SPRI beads purification.

Allow SPRI beads to come to room temperature for at least 30 minutes. Reagents need to be mixed well prior to use and should appear homogeneous and consistent in colour.

- Add the required volume of SPRI beads to the required volume sample (unless otherwise stated 90μl beads: 50μl sample) in a 1.5 ml Eppendorf tube.
- Vortex and leave at room temperature for 5 minutes.
- Place tubes in a magnetic rack.
- Leave for 5 minutes or until sample is clear.
- Carefully remove the clear solution from the tubes and discard.
- Dispense 500µl of 70% ethanol (fresh, not more than 2 days old) into each tube while in the magnetic rack taking care not to disturb the magnetic beads. Aspirate and discard ethanol.
- Repeat the ethanol wash once again (for total of two washes).
- Dry the samples on a heat block (keep the lid of the tube open) at 37°C for 5 to 10 minutes or until the residual ethanol has evaporated (cracks appear in the samples).

- Add specified µl of nuclease free water, vortex and incubate at room temperature for 2 minutes.
- Place tubes into the magnetic rack and leave for 2-3 minutes or until sample is clear.
- Carefully remove the water and retain in a new 1.5ml low bind Eppendorf tube.

7. Appendix 3.

Comparison of time requirements for performing the Standard Amount (SA) library preparation method and the Minimal Starting Amount Capture (MSA-Cap) library preparation method. All time estimations were based on processing 8 samples with single channel pipette.

Step	SA Method	MSA-Cap Method
**Sample fragmentation**	1 hour	1 hour
**End-repair, A-tailing and Ligation (including purifications and quality assessments)**	6.5 hours	5 hours
**Pre-hybridization PCR**	1.5 hours	1.5 hours
**Library Hybridization**	25 hours	25 hours
**Isolation of exomic DNA**	3 hours	2.5 hours

**Post-hybridization PCR**	1.5 hours	1.5 hours
**Bioanalyzer and qPCR quantity assessment**	3.5 hours	3.5 hours
**Flowcell preparation**	2 hours	2 hours
