# Supplementary figures and images for: A Modified Method for Whole Exome Resequencing from Minimal Amounts of Starting DNA
Source: PLoS One. 2012 Mar 5;7(3):e32617. doi: 10.1371/journal.pone.0032617 (PMC3293839; doi:10.1371/journal.pone.0032617)

Figure S1.

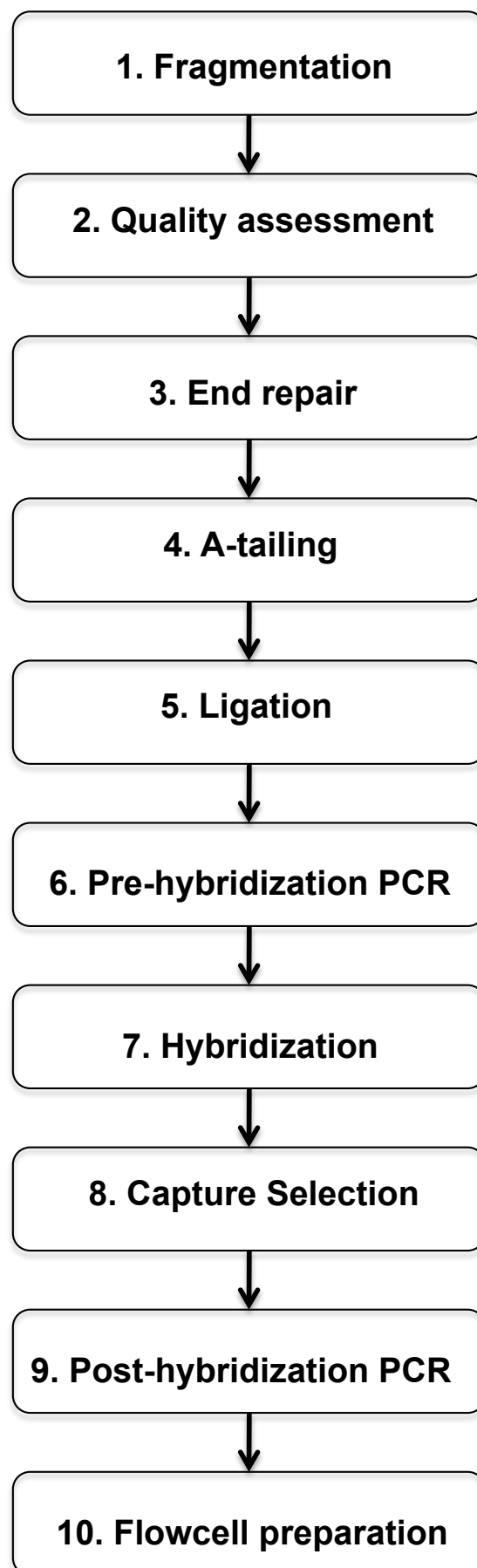

Supplement: Figure S1 — Overview flowchart of the steps involved in target enrichment and sequencing. (PDF) [file pone.0032617.s002.pdf]

Figure S2.

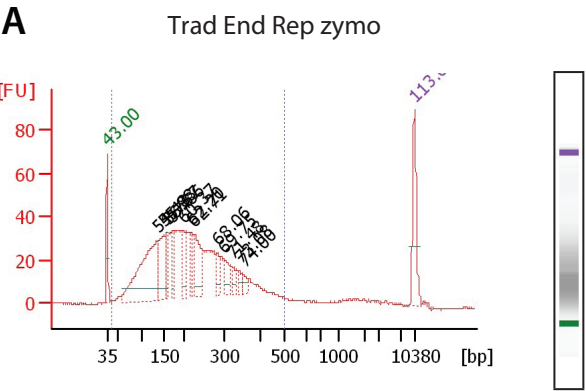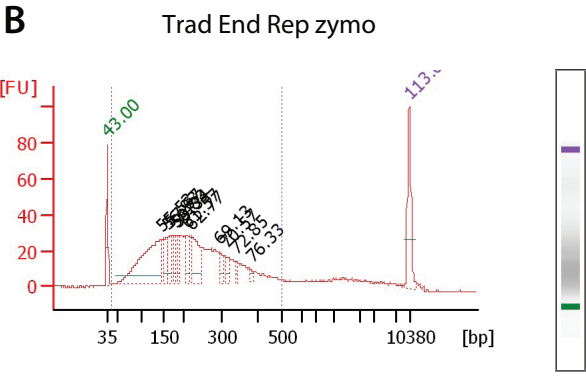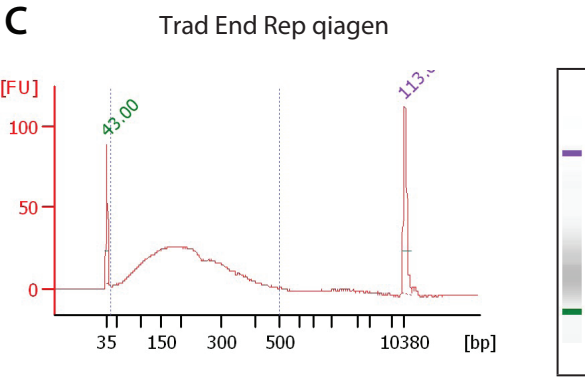

Supplement: Figure S2 — Bioanalyzer High Sensitivity DNA Assay showing extent of recovery of fragmented 50 ng commercial (Novagen) DNA material using Zymo columns (two independent samples in A, B) and Qiagen column (C). The DNA was sheared using the MSA-Cap fragmentation conditions and purified according to manufacturers' instructions. In each case 1 l of the product was loaded. 2.04 ng/µl or 1.69 ng/µl were recovered using Zymo columns (A, B) in contrast to 1.36 ng/µl when using Qiagen column (C). (PDF) [file pone.0032617.s003.pdf]
