# Supplementary material for: A Modified Method for Whole Exome Resequencing from Minimal Amounts of Starting DNA
Source: PLoS One. 2012 Mar 5;7(3):e32617. doi: 10.1371/journal.pone.0032617 (PMC3293839; doi:10.1371/journal.pone.0032617)
Supplement: Table S1 — Sequencing statistics for a PCL-tumour sample, PCL-buccal swab sample and a HapMap (NA12813) sample. (PDF) [file pone.0032617.s004.pdf]

Table S1

	PCL		PCL-buccal Swab		NA12813	
	**SA**	**MSA-Cap**	**SA**	**MSA-Cap**	**SA**	**MSA-Cap**
**Mappable yield**	10.06 Gb	9.43 Gb	10.09Gb	10.26Gb	7.96 Gb	9.31 Gb
**Insert size**	158	167	140	172	157	167
**% PF clusters**	91%	90%	87%	90%	89%	91%
**% Align (PF)**	99%	99%	99%	99%	98%	98%
